# Alcohol and illicit drug use among young people living with HIV compared to their uninfected peers from the Kenyan coast: prevalence and risk indicators

**DOI:** 10.1186/s13011-021-00422-6

**Published:** 2021-11-24

**Authors:** Moses K. Nyongesa, Paul Mwangi, Michael Kinuthia, Amin S. Hassan, Hans M. Koot, Pim Cuijpers, Charles R. J. C. Newton, Amina Abubakar

**Affiliations:** 1 KEMRI-Wellcome Trust Research Programme, Centre for Geographic Medicine Research (Coast), Box 230, Kilifi, Kenya; 2 Department of Clinical, Neuro- and Developmental Psychology, Amsterdam Public Health Research Institute, Vrije Universiteit Amsterdam, Amsterdam, Netherlands; 3 Department of Public Health, Pwani University, Kilifi, Kenya; 4 Department of Psychiatry, University of Oxford, Oxford, UK; 5 Institute for Human Development, Aga Khan University, Nairobi, Kenya

**Keywords:** Substance use, HIV infections, Young people, Prevalence, Risk indicators, Kenya

## Abstract

**Background:**

In sub-Saharan Africa, there is paucity of research on substance use patterns among young people living with HIV (YLWH). To address the gap, we sought to: i) determine the prevalence of substance use, specifically alcohol and illicit drug use, among YLWH compared to their HIV-uninfected peers; ii) investigate the independent association between young people’s HIV infection status and substance use; iii) investigate the risk indicators for substance use among these young people.

**Methods:**

Between November 2018 and September 2019, a cross-sectional study was conducted at the Kenyan coast recruiting 819 young people aged 18–24 years (407 HIV-positive). Alcohol and drug use disorders identification tests (AUDIT and DUDIT) were administered via audio computer-assisted self-interview alongside other measures. Logistic regression was used to determine substance use risk indicators.

**Results:**

The point prevalence of current substance use was significantly lower among YLWH than HIV-uninfected youths: current alcohol use, *13%* vs. *24%, p <  0.01*; current illicit drug use, *7%* vs. *15%, p <  0.01*; current alcohol and illicit drug use comorbidity, *4* vs. *11%, p <  0.01*. Past-year prevalence estimates for *hazardous* substance use were generally low among young people in this setting *(< 10%)* with no significant group differences observed. Being HIV-positive independently predicted lower odds of current substance use, but not *hazardous* substance use. There was overlap of some risk indicators for current substance use between young people with and without HIV including male sex, khat use and an experience of multiple negative life events, but risk indicators unique to either group were also identified. Among YLWH, none of the HIV-related factors was significantly associated with current substance use.

**Conclusions:**

At the Kenyan coast, substance use is a reality among young people. The frequency of use generally appears to be low among YLWH compared to the HIV-uninfected peers. Substance use prevention initiatives targeting young people, regardless of HIV infection status, are warranted in this setting to avert their potential risk for developing substance use disorders, including dependence. The multifaceted intrapersonal and interpersonal factors that place young people at risk of substance use need to be addressed as part of the substance use awareness and prevention initiatives.

**Supplementary Information:**

The online version contains supplementary material available at 10.1186/s13011-021-00422-6.

## Background

The critical developmental phase of adolescence through to young adulthood has been characterized by increases in experimentation and use of – often illicit – substances [1, 2]. Of the substances used by youths, alcohol and marijuana (*Cannabis Sativa*) are the most common [3]. A shift towards high frequency of use of marijuana has particularly been noted among youths globally [2–4]. When alcohol and/or drug use are initiated and continued at a younger age, there is a considerable risk for developing substance use problems presenting as patterns of *hazardous* use or substance dependence [2].

Indeed, studies investigating the prevalence of substance use among youths from both high- and low-income settings report high estimates. A national survey looking at use and abuse of alcohol and illicit drugs among youths in the United States of America (USA) [5] found that 15 and 16% of these youths met the diagnostic criteria for alcohol and drug abuse, respectively, by age 18 years. In sub-Saharan Africa (SSA), the overall prevalence of problematic alcohol and marijuana use among youths is estimated as 33 and 16%, respectively [6]. Substance use is also common among young people living with HIV (YLWH). Cohort studies mainly conducted in high-income settings [7–10] report estimates of substance use in YLWH (mostly alcohol and marijuana) ranging between 13 and 87%. In SSA, research of substance use among YLWH is limited. The few existing studies of which we are aware of [4, 11, 12], report high rates of substance use among YLWH ranging between 18 and 46%.

Substance use disorders (both alcohol and illicit drugs) can have detrimental consequences in the social, economic, behavioral and health aspects of a young person. For instance, it has been linked with increased propensity for risky sexual behaviors such as increased condomless sex or multiple sexual partners [13, 14] predisposing a young person to HIV acquisition risk [12]. Substance use disorder has been found an antecedent to poor educational performance in youths [15]. Substance use disorder also contributes to cases of violence (sexual and physical), delinquency and neglect of social responsibilities [6, 16, 17]. Alcohol misuse and illicit drug use by young people contributes substantially to the global health burden in terms of injuries, morbidity and premature mortality [18]. Moreover, substance use in teenage years is a predictor of its use in adult life [19] with a potential for considerable loss in productivity [16].

For YLWH, the repercussions of substance use on their health outcomes are even greater. There is an impending risk for secondary HIV infection or onward HIV transmission [4, 20] due to risky sexual behavior patterns or sharing of drug-injection needles. Substance use can also result in sub-optimal adherence to antiretroviral therapy (ART) among YLWH [21]. Overtime, this may lead to ART drug resistance [22], immune dysfunction [10], and accelerated HIV disease progression [23].

The literature on risk factors for substance use among the general population of young people is broad [19, 24], owing to: i) the wide variety of substances of abuse that are of different interest to different investigators; and ii) the wide age range of young people (10–24 years) with different studies enrolling young people of varying age groups. In general, these risk factors can be classified as intrapersonal (including biological, demographic, psychological and behavioural factors), interpersonal (relationship with family, peers and other social networks), and structural (including environmental and economic factors) [24, 25]. Intrapersonal factors with the strongest and somewhat consistent evidence of higher risk for substance use among young people include male sex, older age, low self-esteem and psychological problems. Interpersonal risk factors with a degree of consistency across studies involving young people include peer substance use, dysfunctional family interactions (e.g. family conflict, punitive parenting style, parental separation or divorce) and substance use in the family. At the structural level, easy access to substances of abuse, availability of funds, a lack of extensive healthy recreational activities for the youths, and social norms supportive of substance use, especially alcohol use, are some of consistent substance use risk factors among young people [24, 25].

Risk factors for substance use among YLWH are under-investigated. The few cohort studies conducted in Western countries report male sex [7], older youth age group [7], alcohol and marijuana use at home [26], being employed [7], psychological problems [7, 10, 26], history of incarceration [7] and low cluster of differentiation-4 cell count [10] as key risk factors for substance use among YLWH. In SSA, we are only aware of one South African study that has adequately investigated risk indicators for substance use problems among YLWH [11]. According to this study, older age of a youth, male sex and experiencing associative stigma significantly increased the risk of substance use problems among YLWH. A study conducted in Kenya [4] investigating substance use problems among YLWH aged 15–25 years identified male sex, older age, higher level of education, being employed and having a higher income as the significant risk indicators for alcohol use but this was only at the bivariate level of analyses. The authors did not report any multivariate findings.

Substance use remains a major public health concern globally [17, 27] and is now exponentially increasing in resource-limited countries such as those of SSA, more so among young people [6]. Currently, the available body of literature on substance use among young people entails studies mostly from high-income countries. Moreover, most of the existing studies on this topic focus on the general population of young people; there is limited understanding of substance use among special population of youths such as those living with HIV. In SSA where millions of young people live with HIV [28], studies investigating substance use patterns among youths in the context of HIV are scarce. Existing studies from this setting are not only limited in numbers but also characterized by partial analyses, not enrolling an appropriate comparator group, and studying substances of abuse in isolation, largely alcohol use problems. To address these gaps in research, we conducted this study on the Kenyan coast to: i) determine the prevalence of substance use, specifically alcohol and illicit drug use, among YLWH compared to their HIV-uninfected peers; ii) investigate the independent association between young people’s HIV infection status and substance use; iii) investigate the demographic, psychosocial and HIV-related risk indicators for substance use among these young people. To the best of our knowledge, this is the first study from Kenya to compare the burden of substance use between YLWH and their HIV-uninfected peers. Knowledge about the prevalence of substance use among young people, especially those living with HIV, can inform early management approaches that can help avert substance use related negative consequences. Information on risk indicators is also important as it can help identify youths at-risk of substance use problems and inform the type of interventions that might be required for risk reduction.

## Methods

### Study sample

The details of the methodology have been described in another paper [29]. Between November 2018 and September 2019, a cross-sectional study was conducted along the Kenyan coast recruiting young people with and without HIV from HIV clinics and adjacent communities, respectively.

In brief, YLWH were recruited from 20 geographically diverse HIV clinics in Kilifi (*n* = 13) and Mombasa (*n* = 7) counties through consecutive sampling. They were identified from existing clinic records, contacted, and invited for study briefs at a day coinciding with the monthly teen support group meetings. Those who could not be reached via available mobile contacts or without contact details were traced during their next scheduled clinic appointment dates. Eligibility for the study included individuals 18–24 years old, with a documented HIV-positive status, on ART and provision of informed consent for participation. Exclusion criteria included being below 18 years or over 24 years, not providing consent for participation, unverified HIV-positive status and not being on ART. HIV-positive females whose medical records indicated they were pregnant or those who self-reported being pregnant were excluded from participation because pregnancy had the potential of impacting other study outcomes in the larger cross-sectional study as explained elsewhere [29]. Bookings for study interviews were done after participants had been taken through the study in details by research assistants present at the time of the meeting and provided written informed consent. Study interviews were conducted at the HIV clinics in a private and quiet place.

HIV-uninfected peers were recruited from communities adjacent to facilities from where YLWH were recruited. Recruitment was mainly through consecutive sampling following community awareness for the study using posters and flyers. With this recruitment approach, potential participants contacted the study team through the provided contact details in the flyers and posters after which they were invited for study briefing and consenting (at their day of convenience within a week’s time). Participants who provided written informed consent were scheduled for study interviews and HIV self-testing on the same day, one after the other based on their arrival time. Study briefing, consenting, interviews and HIV self-testing (for those who gave informed consent) took place at pitched tents outside the HIV clinics. At any given facility, recruitment of HIV-uninfected young people only commenced after we had completed recruiting YLWH. In Kilifi county, some HIV-uninfected peers were randomly identified and recruited through the already existing Kilifi Health and Demographic Surveillance System (KHDSS) [30]. The KHDSS was used as an additional sampling approach so as to reach the targeted sample size in Kilifi county. Eligibility for HIV-uninfected peers included an age range of 18–24 years, residing in either Kilifi or Mombasa counties, and providing consent for participation including willingness to self-test for HIV using oral self-testing kit (OraQuick) for a confirmation of HIV negative status. Exclusion criteria included age outside 18–24 years, residents of other coastal counties than Kilifi or Mombasa, and not providing consent for participation. Like YLWH, we excluded females who self-reported being pregnant upon enquiry. Participants who were reactive (i.e. tested HIV-positive) were offered counselling by our study counsellor and linked to an HIV facility of their choice for ART initiation.

### Sample size estimation

Sample size calculation was done when designing this study using a two proportions formula based on previously reported prevalence estimates of substance use between YLWH and their HIV-uninfected peers [8]. A total sample of at least 704 participants was required given 85% study power at 5% level of statistical significance. We readjusted this sample size to at least 800 participants in consideration of factors that tend to reduce the final sample size such as non-contact and missing data.

### Measures

Study instruments that do not ask about sensitive personal information were programmed on android tablets using Research Electronic Data Capture (REDCap) platform [31] for face-to-face interviewer administration. Study instruments asking about sensitive information were completed using Audio Computer-Assisted Self-Interview (ACASI) on Windows tablets. Participants had the option of choosing English or the national language (Kiswahili) as their preferred language for answering the questions. ACASI is a preferred platform for improving privacy, confidentiality and reducing social desirability bias when asking personal questions [32].

#### Interviewer administered measures via android tablets

##### Sociodemographic and asset index forms

The sociodemographic form captured participant’s age, sex, area of residence, relationship status, religion, educational level, employment status, living status of parents and whom they currently lived with. The asset index form gathered information about individual or family ownership of disposable items as a proxy indicator of socioeconomic status.

##### Lifestyle and health history form

This form captured data on whether participants currently smoked cigarette or chewed khat using a yes/no response option. Additional information sought from YLWH included disclosure of HIV status, level of satisfaction with current care, clinic accessibility, presence of any current ART side effects, presence of any opportunistic infection and chronic illness (as informed by their clinician).

#### Measures administered via audio computer assisted self-interview (ACASI)

##### Measures assessing substance use

Using a yes/no response option, all the participants were first asked whether they currently used any alcoholic drink (e.g. beer, wine, spirits, vodka, whisky, local alcoholic brews) or any illicit substance of abuse (e.g. marijuana/weed, cocaine, heroin et cetera) to relax, feel better or just to fit in. Then the Alcohol Use Disorders Identification Test (AUDIT) and the Drug Use Disorders Identification Test (DUDIT) were administered to participants responding in the affirmative. The AUDIT is a 10-item self-report scale that screens for patterns of both *hazardous*/harmful alcohol use and alcohol dependence. Item scores are summated to derive a total score, and the maximum total score is 40. A total score of ≥6 and ≥ 8 for females and males respectively, is indicative of *hazardous* drinking (probable alcohol use disorder). A total score of ≥13 or ≥ 15 for females and males respectively, is indicative of probable alcohol dependence [33]. The DUDIT is an 11-item screening instrument for identifying patterns of drug related problems (i.e., *hazardous*/harmful drug use or dependence). Like the AUDIT, item scores are summated with a maximum score being 44. A score of ≥2 and ≥ 6 for females and males respectively, is indicative of problematic drug use. A score of ≥25 for either sex, is indicative of a high probability of drug dependence [34].

##### Measures of emotional problems

The 9-item Patient Health Questionnaire (PHQ-9) [35] and the 7-item Generalized Anxiety Disorder scale (GAD-7) [36] were used to measure depressive and anxiety symptoms, respectively. Items in these measures are scored on a 4-point Likert scale and are then summated to derive a total score that ranges from 0 to 27 for PHQ-9, and 0–21 for GAD-7. The recommended optimal cut-off score of ≥10 for both PHQ-9 and GAD-7 [37, 38] was used to define positive screen for depressive and anxiety symptoms. Presence of an emotional problem was defined as screening positive for both depressive and anxiety symptoms.

##### Negative life events index

A 15-item scale of negative life events was assembled adapting items from the life events questionnaire [39] and from a previous study [40]. Items covered individual-related negative events (e.g. severe illness, lack of basic needs, financial worries), negative events in the domains of school (e.g. quitting school), relationships and love (e.g. infidelity, break-ups), family, close friends and relatives (e.g. bereavement), crime and legal matters (e.g. if ever been robbed or jailed). Respondents were asked to indicate whether they experienced such negative events in the past 1 year on a dichotomous scale (yes/no). A summated score was generated to reflect the total number of life events reported.

#### Clinical records and blood sample (YLWH only)

The following information was extracted from participant medical records and uploaded on the tablets’ electronic data capture platform – most recent height and weight, World Health Organization (WHO) HIV clinical staging, current ART regimen and duration on ART. Blood samples were also collected from all participating YLWH for viral load testing.

### Statistical analysis

All analyses were conducted in STATA version 15.0 (StataCorp LP, College Station, Texas, USA). Frequencies, percentages and means/medians (with standard deviation [SD] or interquartile range [IQR]) were used to summarize sample characteristics. Proportions as percentages were used to estimate prevalence of alcohol and illicit drug use (current use, *hazardous* use and dependence). To investigate the independent association between young people’s HIV infection status and substance use, we used logistic regression analyses adjusting for contextual variables that accounted for differences in substance use. Investigation of substance use risk indicators applied logistic regression models to assess univariate associations between the binary outcome variables (any current alcohol use, illicit drug use and their comorbidity) and exposure variables (demographic, psychosocial, and HIV-related-related factors – for the data from YLWH). Exposure variables having *p*-value < 0.15 in the univariate analysis were entered in the multivariable logistic regression models and forward selection was used to identify best predictive factors. Data were analysed for the whole sample, and separately for YLWH and HIV-uninfected peers. In all the final multivariable models, collinearity diagnostics were performed using STATA’s ‘collin’ syntax and no multicollinearity problems were identified based on an interpretation of the variance inflation factor. Variables in the final models involving the whole sample were interacted by HIV status to check potential effect modification. For all tests of hypothesis, a two-tailed *p*-value of < 0.05 was considered statistically significant, with confidence interval of 95% used to report on precision of the observed estimates.

## Results

Out of 966 young people approached for potential participation (464 HIV-positive youths), 819 were recruited into the study (407 living with HIV and 412 HIV-uninfected peers). Data were analyzed for 812 participants after exclusion of data from seven participants because of missing outcome data (*n* = 2; one HIV-positive youth and one HIV-uninfected youth), refusal to take HIV test (HIV-uninfected youths only) which was the last step of assessment (*n* = 3), or reactive HIV test (*n* = 2). The study recruitment flowchart has been published [29] and is presented here as a supplementary material (see Additional file 1).

### Sample characteristics

Table 1 summarizes the study participants’ characteristics, disaggregated by HIV infection status. Their mean age was 20.9 (SD = 2.1) years, and slightly over-half (50.7%) were females. A slightly higher number of participants resided in the urban setting (50.4%). Near a quarter of the participants had tertiary level of education (23.8%). Most of the participants were either students (37.3%) or unemployed (47.8%). A majority were Christians (74.1%), never married (82.7%) and lived with a family member or relative (88.7%). Close to half (47.2%) of the participants had lost one or both of their parents. In terms of lifestyle habits, 6.2 and 8.9% of the study participants acknowledged smoking cigarette and chewing khat at the time of data collection, respectively.

**Table 1:** Characteristics of young people from the Kenyan coast (*n* = 812), shown by HIV infection status

Characteristic	Whole sample ***N*** = 812	HIV infection status		***P***-value
		HIV uninfected youths, ***n*** = 406	YLWH, ***n*** = 406	
**Area of residence**				
Kilifi (rural)	403 (49.6)	200 (49.3)	203 (50.0)	0.83
Mombasa (urban)	409 (50.4)	206 (50.7)	203 (50.0)	
**Age** – in years as mean (SD)	20.9 (2.1)	21.0 (1.9)	20.8 (2.2)	0.13
**Sex**				
Female	412 (50.7)	182 (44.8)	230 (56.7)	< 0.01
Male	400 (49.3)	224 (55.2)	176 (43.4)	
**Education level**				
*Tertiary*	193 (23.8)	130 (32.0)	63 (15.5)	< 0.01^†^
*Secondary*	354 (43.6)	179 (44.1)	175 (43.1)	
*Primary*	253 (31.2)	93 (22.9)	160 (39.4)	
*None*	12 (1.5)	4 (1.0)	8 (2.0)	
**Employment**				
Formally employed	27 (3.3)	11 (2.7)	16 (3.9)	0.16
Self-employed	94 (11.6)	52 (12.8)	42 (10.3)	
Student	303 (37.3)	162 (39.9)	141 (34.7)	
Unemployed	388 (47.8)	181 (44.6)	207 (51.0)	
**Religion**				
*Muslim*	169 (20.8)	82 (20.2)	87 (21.4)	0.06
*Christian*	602 (74.1)	296 (72.9)	306 (75.4)	
*No religion*	41 (5.1)	28 (6.9)	13 (3.2)	
**Relationship status**, missing = 2				
*Never married*	670 (82.7)	358 (88.6)	312 (76.9)	< 0.01
*Separated with partner*	32 (4.0)	8 (2.0)	24 (5.9)	
*Married/cohabiting*	108 (13.3)	38 (9.4)	70 (17.2)	
**Living arrangement**				
*Family/Relative*	720 (88.7)	359 (88.4)	361 (88.9)	0.91
*Friend/non-relative*	17 (2.1)	8 (2.0)	9 (2.2)	
*Alone*	75 (9.2)	39 (9.6)	36 (8.9)	
**Parental loss**				
*Both parents alive*	429 (52.8)	308 (75.9)	121 (29.8)	< 0.01
*One parent alive*	238 (29.3)	85 (20.9)	153 (37.7)	
*Both parents died*	145 (17.9)	13 (3.2)	132 (32.5)	
***Negative life events***				
*None*	80 (9.9)	51 (12.6)	29 (7.1)	< 0.01
*1–5 events*	476 (58.6)	255 (62.8)	221 (54.4)	
*6+ events*	256 (31.5)	100 (24.6)	156 (38.4)	
**Currently smoking** ^**a**^**,** missing = 1				
*No*	761 (93.8)	374 (92.4)	387 (95.3)	0.08
*Yes*	50 (6.2)	31 (7.6)	19 (4.7)	
**Current khat use,** missing = 1				
*No*	739 (91.1)	364 (89.9)	375 (92.4)	0.21
*Yes*	72 (8.9)	41 (10.1)	31 (7.6)	
***Emotional problems*** ^***#***^				
*Not present*	728 (89.7)	387 (95.3)	341 (84.0)	< 0.01
*Present*	84 (10.3)	19 (4.7)	65 (16.0)	
***Asset index score*** ^***b***^ *– mean (SD)*	2.4 (1.6)	2.6 (1.6)	2.2 (1.6)	< 0.01
***Negative life events scale*** ^***c***^ *– median (IQR)*	4 (2–6)	3 (2–5)	5 (3–7)	< 0.01 ^‡^
***PHQ-9 score*** ^***d***^ *– median (IQR)*	4 (1–9)	3 (1–6)	6 (3–10)	< 0.01 ^‡^
***GAD-7 score*** ^***e***^ *– median (IQR)*	4 (1–7)	3 (0–5)	5 (2–8)	< 0.01 ^‡^

All numbers are reported as frequencies with percentages in brackets unless otherwise specified

*p*-values are for the difference between HIV infected and uninfected youths by sample characteristic

*YLWH* Young people living with HIV, *SD* Standard deviation, *IQR* Interquartile range, *PHQ-9* The 9-item patient health questionnaire, *GAD-7* The 7-item Generalized Anxiety Disorder scale, ^†^ based on Fisher’s exact test, ^‡^ based on Wilcoxon’s ranksum test, ^#^ co-occurrence of both depressive and anxiety symptoms, ^a^ – tobacco-based cigarette, ^b^ – possible score range = 0 to 7, ^c^ – score range = 0 to 15, ^d^ – score range = 0 to 27, ^e^ – score range = 0 to 21

Compared to their HIV-uninfected peers, YLWH were more likely to be female (57% vs. 45%, *p* <  0.01); had lost both parents (33% vs. 3%, *p* <  0.01) and experienced multiple negative life events in the past 1 year (93% vs 87%, *p* <  0.01). Significant differences between the two groups were also observed for educational level and relationship status (*p* <  0.01).

### Participants’ score on study instruments

The scores of the participants on various study instruments are summarized in Table 1. The mean participant score on the asset index was 2.4 (SD = 1.6). The median score on the negative life events scale was 4 (IQR = 2–6). In terms of cumulative negative life events, 10% of the study participants reported not experiencing any negative life event whereas the remaining 90% reported experiencing one or more negative life events in the past 1 year. The median PHQ-9 score was 4 (IQR =1–9) and a similar median (IQR =1–7) was observed for GAD-7 scores. Using a cut-off score of ≥10, 20 and 13% of the participants had depressive and anxiety symptoms, respectively. Co-occurrence of these common mental disorders (herein referred to as presence of emotional problems) was present in 10% (*n* = 84) of the participants.

Compared to HIV-uninfected young people, YLWH were more likely to experience emotional problems, had significantly lower asset index scores on average, and significantly higher negative life events, PHQ-9, and GAD-7 median scores (Table 1).

### HIV-related characteristics of YLWH

HIV-related characteristics of YLWH are presented here as supplementary data for ease of access (see Additional file 2) as these have been reported in detail [29]. In summary, their mean body mass index was within the normal range (mean [SD] = 20.6 [3.6]). Most of these YLWH had been on ART for over 5 years (57.9%), largely first line regimen (81.3%). Over half were in stage 1 of WHO clinical staging of HIV (61.5%) and had viral load *≤*1000 copies/mL (69.0%). More than three-quarters of YLWH were generally satisfied with the current level of care they were receiving (94.3%), had disclosed their HIV-positive status (93.8%), had no current comorbid chronic illness (98.3%), had no current opportunistic infection (93.8%) or medication-related side-effects (73.1%). A quarter of YLWH (25.1%) reported that their present HIV point of care was not easily accessible.

### Prevalence of substance use

Table 2 presents the prevalence estimates for alcohol, illicit drug use and their comorbidity (any current use, *hazardous* use, and probable dependence) in the whole sample and disaggregated estimates by HIV infection status (YLWH vs. HIV-uninfected peers). Group differences in substance use are also compared statistically and presented in this table.

**Table 2:** Prevalence of substance use among YLWH versus HIV-uninfected peers from the Kenyan coast

	Whole sample, ***n*** = 812		HIV uninfected youths, ***n*** = 406		HIV- positive youths, ***n*** = 406		***P***-value ^a^
	Freq.	Prevalence (95% CI)	Freq.	Prevalence (95% CI)	Freq.	Prevalence (95% CI)	
**Point prevalence**							
Any current alcohol use	150	18.5 (15.9, 21.3)	97	23.9 (20.0, 28.3)	53	13.1 (10.1, 16.7)	< 0.001
Any current illicit drug use	90	11.1 (9.1, 13.4)	60	14.8 (11.6, 18.6)	30	7.4 (5.2, 10.4)	< 0.001
Current alcohol & illicit drug comorbidity	58	7.1 (5.6, 9.1)	44	10.8 (8.2, 14.3)	14	3.5 (2.1, 5.7)	< 0.001
**Past-year prevalence**							
Hazardous alcohol use	51	6.3 (4.8, 8.2)	29	7.1 (5.0, 10.1)	22	5.4 (3.6, 8.1)	0.31
Hazardous illicit drug use	58	7.1 (5.6, 9.1)	34	8.4 (6.0, 11.5)	24	5.9 (4.0, 8.7)	0.17
Hazardous alcohol and illicit drug use comorbidity	23	2.8 (1.9, 4.2)	13	3.2 (1.9, 5.4)	10	2.5 (1.3, 4.5)	0.53
Probable alcohol dependence	18	2.2 (1.4, 3.5)	10	2.5 (1.3, 4.5)	8	2.0 (1.0, 3.9)	0.63
Probable illicit drug dependence	4	0.5 (0.2, 1.3)	2	0.5 (0.01, 2.0)	2	0.5 (0.01, 2.0)	1.0

*95% CI* 95% confidence interval; *Freq* Frequency

^a^based on prtest, a two-sample test of differences in proportion using defined binary groups

### Current alcohol or illicit drug use

The overall point prevalence of any current alcohol use was *18.5% (95% CI 15.9, 21.3%)* and that for any current illicit drug use was *11.1% (95% CI 9.1, 13.4%)*. The overall point prevalence of any current alcohol and illicit drug use comorbidity was *7.1% (95% CI 5.6, 9.1%)*. YLWH reported significantly lower frequency of any current alcohol use, illicit drug use, or their co-occurrence than HIV-uninfected peers **(**Table 2**)**.

### *Hazardous* use and probable dependence on alcohol or illicit drugs

The overall past-year prevalence of *hazardous* alcohol and illicit drug use was *6.3% (95% CI 4.8, 8.2%)* and *7.1% (95% CI 5.6, 9.1%)*, respectively. The overall past-year prevalence of the co-occurrence of *hazardous* alcohol and illicit drug use was *2.8% (95% CI 1.9, 4.2%)*. For alcohol and illicit drug dependence, the overall past-year prevalence was *2.2% (95% CI 1.4, 3.5%)* and *0.5% (95% CI 0.2, 1.3%)*, respectively.

Even though the frequency of these substance use problems were slightly lower among YLWH compared to HIV-uninfected peers, the differences were not statistically significant (Table 2).

### Association between HIV infection status and substance use

For any current substance use, we observed that being HIV-positive was significantly associated with lower odds of any current alcohol use (*OR 0.48 95% CI 0.33, 0.69*), illicit drug use (*OR 0.46 95% CI 0.29, 0.73*) and their co-occurrence (*OR 0.29 95% CI 0.16, 0.55*) in the crude logistic regression analyses (see Additional file 3). After adjusting for sex, area of residence, socioeconomic status, religion and exposure to negative life events in the multivariable analyses (see Additional file 3), these associations remained statistically significant: any current alcohol use (*OR 0.47 95% CI 0.32, 0.70*), any current illicit drug use (*OR 0.50 95% CI 0.31, 0.83*) and their co-occurrence (*OR 0.30 95% CI 0.16, 0.58*).

For *hazardous* substance use (also see Additional file 3), the unadjusted odds of *hazardous* alcohol use (*OR 0.74 95% CI 0.42, 1.32*), *hazardous* illicit drug use (*OR 0.69 95% CI 0.40, 1.18*) or their comorbidity (*OR 0.76 95% CI 0.33, 1.76*) were lower with being HIV-positive, but not statistically significant. Adjusting for age, sex, area of residence, socioeconomic status, level of education, employment status, living arrangement, exposure to negative life events and presence of emotional problems, being HIV-positive remained insignificantly associated with lower odds of *hazardous* alcohol (*aOR 0.59 95% CI 0.31, 1.13*), *hazardous* illicit drug use (*aOR 0.59 95% CI 0.32, 1.10*), or their comorbidity (*aOR 0.78 95% CI 0.29, 2.06*).

### Risk indicators for substance use among young people 18–24 years from the Kenyan coast

The small proportion of young people with *hazardous* alcohol and illicit drug use problems limited an analysis of risk indicators for these outcomes since a reduction of range with known effects would be expected hence low precision of effect sizes. Here, we only report the risk indicators for any current substance use.

#### Risk indicators for any current alcohol use

Table 3 and Additional file 4 summarizes these results for the whole sample, and separately for YLWH and HIV-uninfected youths. For YLWH, factors that were significantly associated with higher odds of any current alcohol use (*p <  0.05*) in the univariate analysis included: male sex, being a Christian, having tertiary level of education, living alone, and current cigarette smoking or khat chewing (Table 3). Having no religious affiliation, higher socioeconomic status, viral load >1000copies/mL, presence of emotional problems and opportunistic infection were not significantly associated with any current alcohol use, but the associations were at *p-value <  0.15* (Additional file 4), thus these variables were included in the multivariable modelling. In the final multivariable analysis (Table 3), being a Christian or having no religious affiliation, current khat chewing and presence of emotional problems were significantly associated with higher odds of any current alcohol use. Tertiary level of education was marginally associated with two-fold higher odds of any current alcohol use (*p = 0.05*).

**Table 3:** Logistic regression analysis of risk indicators for any current alcohol use among young people from the Kenyan coast

Covariate	Whole sample, ***n*** = 812		YLWH, ***n*** = 406		HIV uninfected young people, ***n*** = 406	
	Univariate analysis OR (95% CI)	Multivariable analysis aOR (95% CI)	Univariate analysis OR (95% CI)	Multivariable analysis aOR (95% CI)	Univariate analysis OR (95% CI)	Multivariable analysis aOR (95% CI)
**Sex**						
Female	Ref	Ref	Ref	–	Ref	Ref
Male	2.60*** (1.78, 3.78)	1.79** (1.13, 2.85)	2.02** (1.12, 3.63)	–	2.78*** (1.69, 4.60)	1.90** (1.01, 3.57)
**Religion**	Overall *p* = 0.03		Overall *p* = 0.06		Overall *p* = 0.12	
Muslim	Ref	Ref	Ref	Ref	Ref	Ref
Christian	2.01*** (1.20, 3.36)	3.67*** (1.93, 7.00)	2.83** (1.09, 7.36)	4.37*** (1.47, 13.02)	1.71* (0.91, 3.21)	3.54*** (1.53, 8.16)
No religion	2.22* (0.92, 5.35)	4.01** (1.34, 11.97)	4.92* (1.02, 23.76)	8.59** (1.28, 57.53)	1.32 (0.45, 3.86)	2.67 (0.66, 10.83)
**Education**	Overall *p* < 0.01		Overall *p* = 0.03		Overall *p* = 0.02	
*Secondary*	Ref	Ref	Ref	Ref	Ref	Ref
*Tertiary*	2.38*** (1.56, 3.64)	2.42*** (1.47, 4.00)	2.29** (1.10, 4.79)	2.17* (0.97, 4.84)	2.19*** (1.29, 3.73)	2.60*** (1.32, 5.11)
*Primary*	1.00 (0.64, 1.58)	1.08 (0.61, 1.92)	0.87 (0.44, 1.72)	0.61 (0.28, 1.35)	1.34 (0.72, 2.49)	2.04 (0.86, 4.82)
*None*^b^	1.14 (0.24, 5.33)	1.63 (0.25, 10.70)	1.00	1.00	4.59* (0.62, 33.84)	15.42** (1.26, 188.36)
**Living arrangement**	Overall *p* < 0.01		Overall *p* = 0.06		Overall *p* = 0.04	
Family/Relative	Ref	Ref	Ref	–	Ref	Ref
Friend/non-relative	1.52 (0.49, 4.75)	1.22 (0.33, 4.51)	2.17 (0.44, 10.79)	–	1.18 (0.23, 5.97)	0.55 (0.06, 4.66)
Alone	2.48*** (1.47, 4.16)	2.45*** (1.30, 4.61)	2.53** (1.11, 5.75)	–	2.47** (1.24, 4.89)	3.48*** (1.50, 8.05)
**Asset index**	1.27*** (1.14, 1.42)	1.34*** (1.17, 1.55)	1.16* (0.97, 1.38)	–	1.32*** (1.14, 1.53)	1.44*** (1.18, 1.76)
**Negative life events**	Overall *p* < 0.01				Overall *p* < 0.01	
None	Ref	Ref	–	–	Ref	Ref
1–5 events	1.27 (0.62, 2.57)	1.35 (0.59, 3.09)	–	–	1.61 (0.69, 3.78)	1.47 (0.53, 4.09)
6+ events	2.48** (1.21, 5.09)	3.17*** (1.35, 7.46)	–	–	3.85*** (1.58, 9.42)	4.13** (1.37, 12.45)
**Currently smoking**						
No	Ref	Ref	Ref	–	Ref	Ref
Yes	10.68*** (5.76, 19.81)	5.97*** (2.73, 13.08)	5.53*** (2.11, 14.47)	–	17.48*** (6.91, 44.18)	13.42*** (4.45, 40.50)
**Current khat use**						
No	Ref	Ref	Ref	Ref	Ref	Ref
Yes	10.81*** (6.40, 18.24)	8.59*** (4.46, 16.55)	11.43*** (5.21, 25.10)	15.75*** (6.51, 38.12)	10.52*** (5.11, 21.67)	7.99*** (3.11, 20.51)
**Emotional problems**^a^						
Not present	–	–	Ref	Ref	–	–
Present	–	–	1.88* (0.94, 3.76)	2.27** (1.02, 5.05)	–	–
**n of the final model**		811		398		405
**Variance explained (R**^**2**^**)**		25.0%		17.6%		30.1%

* *p* value< 0.15, ** *p* value < 0.05, *** *p* value < 0.01

Only *variables* with *p*-value < 0.15 in the univariate and *p* < 0.05 in the multivariable analysis are presented here

^a^co-occurrence of both depressive and anxiety symptoms

^b^for this variable category, no participant currently used any alcohol type, hence there was perfect prediction of failure in the regression analyses denoted by null value of 1.00

*OR* Odds ratio, *aOR* Adjusted odds ratio, *Ref* Reference group

For HIV-uninfected young people, increasing age, male sex, urban residence, having tertiary level of education, living alone, higher socioeconomic status, experiencing multiple negative life events (6 or more events relative to none), having only one parent alive, and current cigarette smoking or khat chewing were the factors that were significantly associated with higher odds of any current alcohol use (*p <  0.05*) in the univariate analysis (Table 3 and Additional file 4). Being a Christian, having no formal education and death of both parents were not significantly associated with any current alcohol use, but the associations were at *p-value <  0.15* (Additional file 4), thus these variables were included in the multivariable modelling. In the final multivariable analysis (Table 3), male sex, being a Christian, having tertiary or no level of education, living alone, higher socioeconomic status, experiencing multiple negative life events (6 or more events), and current cigarette smoking or khat chewing were significantly associated with higher odds of any current alcohol use.

Under the univariate logistic regression analysis involving the whole sample, factors that were significantly associated with higher odds of any current alcohol use (*p <  0.05*) included: increasing age, male sex, urban residence, being a Christian, having tertiary level of education, living alone, higher socioeconomic status, experiencing multiple negative life events (6 or more events relative to none), and current cigarette smoking or khat chewing (Table 3 and Additional file 4). Having no religious affiliation was not significantly associated with any current alcohol use, but the association was at *p-value <  0.15* (Additional file 4), thus this variable was included in the multivariable modelling. In the final multivariable analysis (Table 3), male sex, being a Christian or having no religious affiliation, tertiary level of education, living alone, higher socioeconomic status, experiencing multiple negative life events (6 or more events), and current cigarette or khat chewing were significantly associated with higher odds of any current alcohol use. Exploring the interaction between each of these risk indicators and HIV infection status in the multivariable model, there was a statistically significant interaction between smoking and HIV infection status (*p = 0.009*) indicating that the main effect of smoking on alcohol use – controlling for other independent variables – is significantly stronger for HIV-uninfected youths than for YLWH (Table 3).

#### Risk indicators for any current illicit drug use

Table 4 and Additional file 4 summarizes these results for the whole sample, and separately for YLWH and HIV-uninfected youths. For YLWH, factors that were significantly associated with higher odds of any current illicit drug use (*p <  0.05*) in the univariate analysis included: male sex, urban residence, being a Muslim and current use of alcohol (Table 4). Not being married and living alone were not significantly associated with any current illicit drug use, but the associations were at *p-value < 0.15* (Additional file 4), thus these variables were included in the multivariable modelling. None of the HIV-related factors were associated with any current illicit drug use at *p-value < 0.05 or < 0.15* (Additional file 4). In the final multivariable analysis (Table 4), male sex, urban residence, being a Muslim and current use of alcohol remained significantly associated with higher odds of any current illicit drug use.

**Table 4:** Logistic regression analysis of risk indicators for any current illicit drug use among young people from the Kenyan coast

Covariate	Whole sample, ***n*** = 812		YLWH, ***n*** = 406		HIV uninfected young people, ***n*** = 406	
	Univariate analysis OR (95% CI)	Multivariable analysis aOR (95% CI)	Univariate analysis OR (95% CI)	Multivariable analysis aOR (95% CI)	Univariate analysis OR (95% CI)	Multivariable analysis aOR (95% CI)
**Sex**						
Female	Ref	Ref	Ref	Ref	Ref	Ref
Male	8.07*** (4.32, 15.09)	6.65*** (3.35, 13.20)	5.89*** (2.35, 14.76)	4.80*** (1.82, 12.68)	9.32*** (3.91, 22.23)	13.08*** (4.28, 39.98)
**Area of residence**						
Rural (Kilifi)	Ref	Ref	Ref	Ref	Ref	–
Urban (Mombasa)	2.14*** (1.35, 3.39)	2.12** (1.19, 3.78)	3.58*** (1.50, 8.54)	2.93** (1.14, 7.54)	1.68* (0.96, 2.95)	–
**Religion**	Overall *p* = 0.02		Overall *p* = 0.12		Overall *p* = 0.14	
*Muslim*	1.77** (1.07, 2.92)	2.45*** (1.30, 4.61)	2.32** (1.05, 5.11)	3.13** (1.24, 7.89)	1.52 (0.79, 2.93)	–
*Christian*	Ref	Ref	Ref	Ref	Ref	–
*No religion*	2.36** (1.04, 5.37)	2.75 (0.91, 8.32)	1.33 (0.16, 10.83)	0.68 (0.06, 7.42)	2.26* (0.90, 5.68)	–
**Asset index**	1.25*** (1.09, 1.43)	–	–	–	1.44*** (1.20, 1.72)	1.49*** (1.15, 1.92)
**Negative life events**	Overall *p* = 0.06				Overall *p* = 0.08	
None	Ref	Ref	–	–	Ref	–
1–5 events	3.15* (0.96, 10.33)	3.32 (0.91, 12.10)	–	–	2.72* (0.80, 9.17)	–
6+ events	4.06** (1.22, 13.59)	4.08** (1.08, 15.48)	–	–	4.00** (1.13, 14.17)	–
**Parent living status**	Overall *p* = 0.05				Overall *p* = 0.01	
Both alive	2.26** (1.04, 4.89)	3.15** (1.31, 7.58)	–	–	Ref	Ref
One alive	2.66** (1.19, 5.95)	3.38*** (1.35, 8.49)	–	–	2.26*** (1.25, 4.11)	3.04*** (1.35, 6.81)
Both dead^b^	Ref	Ref	–	–	1.00	1.00
**Current alcohol use**						
No	Ref	Ref	Ref	Ref	Ref	Ref
Yes	12.41*** (7.65, 20.14)	13.32*** (7.56, 23.48)	7.56*** (3.43, 16.66)	9.90*** (3.93, 24.95)	15.20*** (8.00, 28.91)	11.92*** (5.84, 24.32)
**Emotional problems**^a^						
Not present	–	–	–	–	Ref	Ref
Present	–	–	–	–	2.85** (1.04, 7.81)	7.30*** (1.65, 32.30)
**n of the final model**		812		406		393
**Variance explained (R**^**2**^**)**		32.3%		23.3%		38.8%

* *p* value< 0.15, ** *p* value < 0.05, *** *p* value < 0.01

Only *variables* with *p*-value < 0.15 in the univariate and *p* < 0.05 in the multivariable analysis are presented here

^a^co-occurrence of both depressive and anxiety symptoms

^b^for this variable category, no participant currently used any illicit drug, hence there was perfect prediction of failure in logistic regression analyses denoted by null value of 1.00

*OR* Odds ratio, *aOR* Adjusted odds ratio, *Ref* Reference group

For HIV-uninfected young people, the following factors were significantly associated with higher odds of any current illicit drug use (*p < 0.05*) in the univariate analysis: male sex, higher socioeconomic status, experiencing multiple negative life events (6 or more events relative to none), having only one parent alive, current use of alcohol and presence of emotional problems (Table 4). Urban residence, experiencing multiple negative life events (up to 5 events relative to none) and having no religious affiliation were not significantly associated with any current illicit drug use, but the associations were at *p-value < 0.15 (*Table 4 and Additional file 4), thus these variables were included in the multivariable modelling. In the final multivariable analysis (Table 4), male sex, higher socioeconomic status, having one parent alive, current use of alcohol and presence of emotional problems remained significantly associated with higher odds of any current illicit drug use.

Factors that were significantly associated with higher odds of any current illicit drug use (*p < 0.05*) in the univariate logistic regression analysis involving the whole sample included: male sex, urban residence, being a Muslim or having no religious affiliation, higher socioeconomic status, experiencing multiple negative life events (6 or more events relative to none), having one or both parents alive and current use of alcohol (Table 4). Not being married and experiencing multiple negative life events (up to 5 events relative to none) were not significantly associated with any current illicit drug use, but the associations were at *p-value < 0.15* (Additional file 4), thus these variables were included in the multivariable modelling*.* In the final multivariable analysis (Table 4), male sex, urban residence, being a Muslim, experiencing multiple negative life events (6 or more events), having one or both parents alive and current use of alcohol remained significantly associated with higher odds of any current illicit drug use. Exploring the interaction between each of these risk indicators and HIV infection status in the multivariable model, there was a statistically significant interaction between parental living status and HIV infection status (*p = 0.02*) indicating that the main effect of parent living status on illicit drug use – controlling for other independent variables – is only significant for the HIV-uninfected group (Table 4).

#### Risk indicators for any current alcohol and illicit drug use comorbidity

Table 5 and Additional file 4 summarizes these results for the whole sample, and separately for YLWH and HIV-uninfected youths. For YLWH, factors that were significantly associated with higher odds of any current alcohol and illicit drug use comorbidity (*p < 0.05*) in the univariate analysis included: male sex, living alone, an experience of multiple negative life events (6 or more events), and presence of opportunistic infection (Table 5 and Additional file 4)**.** Being formally employed, self-employed or unemployed, on second line ART, and unsatisfied with current HIV care were not significantly associated with any current alcohol and illicit drug use comorbidity but the associations were at *p-value < 0.15* (Additional file 4), hence these variables were included in the multivariable modelling. None of the HIV-related factors were associated with any current alcohol and illicit drug use at *p-value < 0.05 or < 0.15* (Additional file 4). In the final multivariable analysis (Table 5), male sex and experiencing multiple negative life events (6 or more events) remained significantly associated with higher odds of any current alcohol and illicit drug use comorbidity.

**Table 5:** Logistic regression analysis of risk indicators for current alcohol and illicit drug use comorbidity among young people from the Kenyan coast

Covariate	Whole sample, ***n*** = 812		YLWH, ***n*** = 406		HIV uninfected young people, ***n*** = 406	
	Univariate analysis OR (95% CI)	Multivariable analysis aOR (95% CI)	Univariate analysis OR (95% CI)	Multivariable analysis aOR (95% CI)	Univariate analysis OR (95% CI)	Multivariable analysis aOR (95% CI)
**Sex**						
Female	Ref	Ref	Ref	Ref	Ref	Ref
Male	7.21*** (3.37, 15.43)	9.40*** (4.25, 20.80)	3.40** (1.05, 11.04)	4.29** (1.30, 14.21)	9.67*** (3.39, 27.60)	14.12*** (4.58, 43.53)
**Education**	Overall *p* = 0.14					
*Secondary*	Ref	Ref	–	–	–	–
*Tertiary*	1.72* (0.89, 3.33)	1.62 (0.79, 3.31)	–	–	–	–
*Primary*	1.43 (0.75, 2.72)	2.66*** (1.31, 5.43)	–	–	–	–
*None*^a^	1.00	1.00	–	–	–	–
**Asset index**	1.33*** (1.13, 1.57)	1.46*** (1.21, 1.77)	–	–	1.47*** (1.19, 1.80)	1.80*** (1.39, 2.33)
**Negative life events**	Overall *p* = 0.01		Overall *p* = 0.01		Overall *p* = 0.03	
None^a^	Ref	Ref	1.00	1.00	Ref	Ref
1–5 events	5.13* (0.69, 38.17)	5.98 (0.78, 45.60)	Ref	Ref	5.68* (0.75, 42.82)	6.52 (0.81, 52.09)
6+ events	9.70** (1.30, 72.48)	17.33*** (2.23, 134.67)	5.51** (1.51, 20.10)	6.44*** (1.74, 23.83)	10.24** (1.32, 79.32)	17.40*** (2.04, 148.07)
**Parent living status**	Overall *p* = 0.10				Overall *p* = 0.01	
Both alive	2.41* (0.92, 6.28)	3.72** (1.34, 10.30)	–	–	Ref	Ref
One alive	2.43* (0.89, 6.65)	3.35** (1.16, 9.70)	–	–	2.32** (1.19, 4.52)	2.94*** (1.34, 6.41)
Both dead^a^	Ref	Ref	–	–	1.00	1.00
**n of the final model**		800		377		393
**Variance explained (R**^**2**^**)**		19.1%		12.4%		24.6%

* *p* value< 0.15, ** *p* value < 0.05, *** *p* value < 0.01

Only *variables* with *p*-value < 0.15 in the univariate and *p* < 0.05 in the multivariable analysis are presented here

^a^for these variable categories, no participant had any current alcohol and illicit drug use comorbidity, hence there was perfect prediction of failure in logistic regression analyses denoted by null value of 1.00

*OR* Odds ratio, *aOR* Adjusted odds ratio, *Ref* Reference group

For HIV-uninfected young people, the following factors were significantly associated with higher odds of any current alcohol and illicit drug use comorbidity (*p < 0.05*) in the univariate analysis: male sex, higher socioeconomic status, experiencing multiple negative life events (6 or more events) and having only one parent alive (Table 5). Experiencing multiple negative life events (up to 5 events) was not significantly associated with any current alcohol and illicit drug use comorbidity, but the association was at *p-value < 0.15* (Table 5). In the final multivariable analysis (Table 5), male sex, higher socioeconomic status, experiencing multiple negative life events (6 or more events) and having one parent alive remained significantly associated with higher odds of any current alcohol and illicit drug use comorbidity.

Under the univariate logistic regression analysis involving the whole sample, factors that were significantly associated with higher odds of any current alcohol and illicit drug use comorbidity (*p < 0.05*) included: male sex, higher socioeconomic status, and experiencing multiple negative life events (6 or more events; Table 5). Having no religious affiliation, tertiary level of education, one or both parent alive and experiencing multiple negative life events (up to 5 events) were not significantly associated with any current alcohol and illicit drug use comorbidity, but the associations were at *p-value < 0.15* (Additional file 4), thus these variables were included in the multivariable modelling. In the final multivariable analysis (Table 5), male sex, primary level of education, higher socioeconomic status, experiencing multiple negative life events (6 or more events) and having one or both parent alive were significantly associated with higher odds of any current alcohol and illicit drug use comorbidity. When exploring for potential interactions, we found statistically significant interaction between HIV infection status and both socioeconomic status (*p = 0.012*) and parental living status (*p = 0.004*) indicating that the main effect of socioeconomic status and parent living status on comorbid alcohol and illicit drug use – controlling for other independent variables – are only significant for the HIV-uninfected group (Table 5).

## Discussion

This study is among the first few describing substance use, specifically alcohol and illicit drug use, in YLWH compared to HIV-uninfected peers at the coast of Kenya. The specific study objectives were to determine the prevalence of substance use, investigate the independent association between young people’s HIV infection status and substance use and identify substance use risk indicators. On the Kenyan coast, we report relatively high point prevalence of current alcohol use (19%) and illicit drug use (11%) among young people aged 18–24 years. The frequency of current use of alcohol, illicit drugs or both was significantly lower among YLWH compared to HIV-uninfected peers. The past-year prevalence of *hazardous* alcohol, illicit drug use and their co-occurrence, and substance use dependence were low (< 10%) in this sample and there were no significant differences observed between YLWH and HIV-uninfected peers. Being HIV-positive independently predicted lower odds of current substance use but not *hazardous* use. Overall, study findings suggest that certain demographic and psychosocial factors significantly increase the odds of current substance use among young people with or without HIV in this setting, some overlapping between youths with and without HIV for instance khat use, male sex and experience of multiple negative life events; while others were unique to either group. Among YLWH, none of the HIV-related factors were significantly associated with current substance use in any of the final multivariable models. The observed main effects of the risk indicators in the analysis involving the whole sample should be interpreted cautiously as there were significant interactions between some factors (cigarette smoking, socioeconomic status and parental living status) and HIV infection status.

The point prevalence estimates for current substance use reported in this study compares to what has been reported in the literature for recent substance use among both YLWH [26] and young people in the general population [6, 41, 42] but is way higher than the national estimates [43]. The reported past-year prevalence estimates for *hazardous* substance use in the whole sample and either study groups (YLWH and HIV-uninfected peers) are way lower than what has previously been reported among young people in another Kenyan setting [4], other SSA settings [11, 12, 44] and elsewhere [7, 8]. Our estimates of *hazardous* substance use are also lower compared to the estimates reported from a national rapid situation assessment of the status of substance use disorder in Kenya [43]. There are three potential explanations as to why the prevalence estimates from this study may be different from what has been reported previously. First, *hazardous* substance use may indeed be low in this highly literate youthful population from the Kenyan coast (67% with secondary and above level of education). Most of the young people in this study may have been well-informed about the harmful effects of substance use on the different spheres of their life either through self-education or as part of their education curricula. Second, due to concerns of family, societal or legal backlash for alcohol and drug abuse, there may have been a reporting bias despite the use of ACASI technology and assurance about confidentiality of responses. Differences in age groups of young people being studied, the study setting, information source (self-report vs. other informants or toxicology screening) and measurement tools used across studies may be the third alternative explanation. Regardless, our finding supports the observation that alcohol and illicit drug use are a reality among young people in Kenya [4, 45]. Since this is the only study that compares substance use between YLWH and their uninfected peers in the Kenyan context, we recommend more research on this topic involving young people with and without HIV, preferably from the coast of Kenya or a similar setting, to compare findings.

In general, we observed a lower proportion of substance use among YLWH than HIV-uninfected peers. A similar trend has been noted in other previous researches involving young people [10, 46]. In contrast, other studies [8, 26] report a higher proportion of substance use among YLWH than their HIV-uninfected peers. In this study, the unadjusted and adjusted odds of both current and *hazardous* substance use were consistently lower among YLWH compared to their HIV-uninfected peers. However, statistical differences were only observed for current substance use but not *hazardous* use. In the adjusted analyses, HIV-positive status was significantly associated with a 51, 54 and 71% less likelihood of any current alcohol use, illicit drug use, and their co-occurrence, respectively. This finding contrasts that by Alperen et al. [26]. Comparing recent substance use among perinatally HIV-infected youths vs. perinatally exposed but uninfected youths, these authors did not find any significant group differences by HIV infection status. A potential explanation for our finding is that during their clinic visits, YLWH are more likely to be receiving health information offered during teen/peer meetings on, for example, the negative interactions between substance use and health outcomes including ART adherence [21], and their uptake of such information mitigates the risk for substance use. The finding that HIV infection status is not a significant predictor of *hazardous* substance use compares to previous research [8, 10].

In the analysis involving the whole sample, several young people’s demographic and psychosocial factors were significantly associated with current substance use including participants’ sex, religion, level of education, experience of multiple negative life events, socioeconomic and parent living status. Additionally, current use of other non-illicit drugs like tobacco-based cigarettes or khat was significantly associated with any current alcohol use while any alcohol use was significantly associated with any current illicit drug use. Even though these findings are consistent with what has been reported in the literature [24, 25, 42, 47], the statistically significant interactions between HIV infection status and risk indicators like cigarette smoking, socioeconomic status and parental living status in the multivariable analyses limits any meaningful interpretation of the observed main effects. Also, the fact that the main effect of some variables was only sustained in the whole sample analysis but not in the separate sample analysis points towards a potential for effect modification. For these reasons, the findings disaggregated by HIV infection status are more appropriate for interpretation. A separate study with an objective of quantifying the interaction effect of HIV infection status in young people exposed to important risk factors for substance use in this or a similar setting is needed; this was beyond the scope of the present study. Findings from such an enquiry will be important in informing the need for prioritization of intervention or care considering that over three-quarters of young people living with HIV are in sub-Saharan Africa [28] and the negative consequences of substance use are more pronounced in YLWH [26].

In the separate analyses of data from YLWH and HIV-uninfected peers, there was a substantial overlap of risk indicators for current substance use, an observation that is also reported by Alperen et al. [26]. Khat chewing and being affiliated to Christianity were the risk indicators common to both groups of young people for any current alcohol use. Risk indicators common to both groups for any current illicit drug use were male sex and current use of alcohol. Risk indicators common to both groups for any current alcohol and illicit drug use comorbidity were male sex and an experience of multiple negative life events. Male sex has been found a consistent risk indicator for any substance use across studies involving YLWH [7, 11] or the general population of young people [24, 25, 48]. In the literature, an association between substance use and stressful life events has also been established among youths [24] including those living with HIV [26].

The observed association between khat chewing and higher odds of alcohol use in this study is supported by findings from previous research [49, 50] and so is the association between any alcohol use and higher odds of illicit drug use [47]. The finding that affiliation to Christianity was associated with higher odds of any current alcohol use is in the expected direction as the reference category was being Muslim. Islamic practices are highly deterrent of alcohol use; therefore, this group is likely to be minimally predisposed to the risk of alcohol use. However, this finding should not be interpreted at face value because of two reasons. First, Christianity is broadly split into three: Catholic, Protestant and Orthodox. Based on how data was collected, our analyses cannot identify the exact group(s) at elevated risk of alcohol use. Second, unlike other studies [41], we did not grade the degree of religious practice (low vs. high religiosity). Francis et al. [41] found that high religiosity was associated with lower odds of alcohol and other drug use. Nevertheless, the findings from this study show the important role that religious organizations can have in the prevention of substance use among young people subscribing to some form of religion.

Risk indicators unique to either YLWH or HIV-uninfected group were also identified, as has been observed in another research work [26]. Higher socioeconomic status was a unique risk indicator for any current alcohol use, illicit drug use or their comorbidity among HIV-uninfected young people supporting the finding that availability of funds facilitates young people’s substance use [45]. Other unique risk indicators for any current substance use among HIV-uninfected young people included low education, living alone, current cigarette smoking and having one parent alive. Male sex and an experience of multiple negative life events also emerged as unique to HIV-uninfected peers specifically for any current alcohol use. Low education and cigarette smoking have been identified as risk indicators for substance use in the general population of young people [25, 44]. In the absence or reduced monitoring from parents or guardians, young people can begin to experiment with substances mostly as a result of peer influence [51]. Among YLWH, the unique risk indicators specifically for current illicit drug use included urban residence and being a Muslim. Mombasa, an urban setting and a transit hub for seafarers, is well-known for easy access to illicit drugs with many people who use drugs engaging in risky large-scale sex trade to raise funds for sustaining their drug use habits [45, 52], an observation that may partly explain our finding. As previously mentioned, caution should be taken when interpreting the finding on religious affiliation and substance use as we did not determine the religiosity level among study participants.

A unique risk indicator of greatest public health concern was the presence of internalizing mental health problems (depressive and anxiety symptoms) which was significantly associated with any current alcohol use among YLWH and any current illicit drug use among HIV-uninfected young people. In the literature, the association between substance use and mental health problems in young people with or without HIV has been documented [10, 26, 53]. The cause-effect relationship between substance use and mental health problems cannot be established with the study design employed in this work. Therefore, we call for additional studies to build on this finding by employing study designs that will allow causal inference. Our analyses, however, support the need to address, early enough, young peoples’ mental health issues since substance use in combination with psychiatric problems can lead to risky sexual behaviours [10] placing youths at risk of worse outcomes, for example HIV acquisition (new strains for YLWH, potentially virulent).

Among YLWH, this study found no significant associations between current substance use and any of the HIV-related factors including a measure of disease severity (HIV viral load). Similar findings have been reported in the literature [7, 54]. It is possible that the lack of significant associations between substance use and HIV-related factors in this study was because these young people are already engaged in care, and almost all (94%) were satisfied with the current quality of care they were receiving. People living with HIV may engage in substance use as a coping mechanism to living with a lifetime infection and outcomes of its treatment, especially if they are not receiving satisfactory medical care [10].

The findings reported in this study should be interpreted within several study limitations. First, the potential for social desirability bias in responding to substance use questions cannot be underplayed. The use of ACASI may have mitigated this bias, but we cannot completely rule out social desirability since the measures we used are based on subjective self-report, hence a possibility of outcome underreporting. Additionally, the measure we used to assess illicit drug use – the DUDIT – does not specifically detail individual drug types, therefore we are unable to provide the prevalence estimates for individual drug types. A follow-up study is encouraged to provide data on individual illicit drug use rates among young people at the Kenyan coast. Furthermore, the cross-sectional study design limits any causal inference for any significant associations observed in this study. Relatedly, because the proportion of youths with *hazardous* substance use was particularly low, we were unable to investigate its associated risk indicators. An understanding of risk indicators for problematic substance use would have better informed intervention efforts in the clinical setting considering that in settings such as Kenya, resources are limited and need to be judiciously distributed for the many competing priorities. We did not collect and analyse data on family history of substance use or peer influence which have been highlighted as important risk indicators for substance use among young people [24, 26]. This was a non-probability sample of young people, and participating YLWH were already engaged in medical care in public facilities. The generalizability of the findings to the broader population of youths is limited, including young people with HIV out of care or seeking care from private facilities.

### Implications of the study findings for policy, practice and future research

Despite the above stated limitations, this work has important implications for policy, practice and future research related to substance use among young people in their early adulthood. We found a relatively high prevalence of current alcohol and illicit drug use among the sampled young people, higher than the national estimates [43]. At the Kenyan coast, there is a need for initiating substance use prevention programmes targeting all young people, regardless of their HIV infection status, to prevent those reporting any current substance use from potentially progressing to develop substance use disorders, including substance dependence.

The prevalence of current alcohol and illicit drug use was significantly lower among YLWH compared to the HIV-uninfected young people. This finding contrasts reports from studies conducted in other settings [8, 26]. Compared to the national estimate [43], we also observed a lower prevalence of *hazardous* substance use among young people at the Kenyan coast, more so among those living with HIV. Considering that this study is among the first few from SSA, and the first from the Kenyan coast to study substance use patterns among young people, more studies on this topic (preferably of comparative design) are needed to compare our findings, further understand the burden of substance use and substance use disorders among young people living in a setting with the greatest burden of HIV, and to evaluate the need for screening for substance use disorders in these youthful sub-population at different structural levels, including the HIV clinics.

In this study, we identified several factors that were significantly associated with current substance use among young people at the Kenyan coast. However, inference on causality is limited due to the employed cross-sectional study design. Therefore, we recommend future studies to examine further the reported associations using study designs that can allow causal inferences, including temporality of the associations and dose-response relationships.

We observed significant interactions between HIV infection status and several risk indicators for current substance use like cigarette smoking, socioeconomic and parental living statuses. Since this study’s objectives were not centred around effect modification, a separate study specifically examining youth’s HIV infection status as an effect modifier of the association between substance use and these exposure variables is needed in this or a similar setting.

## Conclusions

Despite the above-mentioned limitations, important conclusions can be drawn from this work. The study found a relatively high point prevalence of any current alcohol and illicit drug use among young people from the Kenyan coast, but interestingly, significantly lower among YLWH than the HIV-uninfected youths. The past-year prevalence of *hazardous* substance use among young people in this setting appears to be low, and the frequency of use is not any different by youths’ HIV infection status. Substance use prevention initiatives targeting young people at the Kenyan coast, regardless of HIV infection status, are much needed. This is particularly important because there is a considerable risk for developing substance use disorders, including dependence, when substance use is initiated and continued at a younger age [2, 7]. A better understanding of the different drug types used by the young people can properly guide the prevention efforts. We found that HIV infection status independently predicts lower odds of current substance use, but not *hazardous* substance use. Additionally, certain demographic and psychosocial factors were significantly associated with higher odds of current substance use, some shared across youths with and without HIV while others were unique to either group. Overall, these results underline the impetus of addressing the multifaceted intrapersonal and interpersonal factors that place young people at risk of substance use as part of substance use awareness and prevention initiatives.

## Supplementary Information

**Additional file 1.** Participant recruitment process. This additional file shows a flow diagram of the comprehensive participant recruitment process for both young people living with HIV and their uninfected peers from the community alongside explanatory notes.

**Additional file 2.** HIV-related characteristics of YLWH. This additional file summarizes, in a table, the HIV-related characteristics of 406 young people living with HIV aged 18–24 years from the Kenyan coast.

**Additional file 3.** Association between HIV-infection status and current substance use. This additional file summarizes, in a table, the association between HIV infection status and substance use (alcohol, illicit drugs, or both) among young people from the Kenyan coast.

**Additional file 4.** Univariate analysis of risk indicators for current substance use among young people. This additional file summarizes, in a table, results from univariable logistic regression analyses of the risk indicators for current alcohol use, illicit drug use, or both, among young people from the Kenyan coast.

## Data Availability

The dataset and associated files used for analysis of this study are available in Harvard dataverse at 10.7910/DVN/4XMXEI. Application for access can be made through the data governance committee of the KEMRI Wellcome Trust Research Programme who will review the application and advise as appropriate ensuring that uses are compatible with the consent obtained from participants for data collection. Requests can be sent to the coordinator of the Data Governance Committee using the following email: dgc@kemri-wellcome.org

## Abbreviations

ACASI: Audio computer assisted self-interview
ART: Antiretroviral therapy
AUDIT: Alcohol Use Disorders Identification Test
DUDIT: Drug Use Disorders Identification Test
GAD-7: 7-Item generalized anxiety disorder scale
KHDSS: Kilifi health and demographic surveillance system
PHQ-9: 9-item patient health questionnaire
REDCap: Research electronic data capture
SSA: Sub-Saharan Africa
USA: United States of America
WHO: World Health Organization
YLWH: Young People Living with HIV
