# Supplementary material for: Alcohol and illicit drug use among young people living with HIV compared to their uninfected peers from the Kenyan coast: prevalence and risk indicators
Source: Subst Abuse Treat Prev Policy. 2021 Nov 24;16:86. doi: 10.1186/s13011-021-00422-6 (PMC8613997; doi:10.1186/s13011-021-00422-6)
Supplement: Supplementary file 1 — Additional file 1. Participant recruitment process. This additional file shows a flow diagram of the comprehensive participant recruitment process for both young people living with HIV and their uninfected peers from the community alongside explanatory notes. [file 13011_2021_422_MOESM1_ESM.docx]

**Additional File 1: Participant recruitment process and explanatory notes.**

Sample analyzed

n=406

**YLWH**

Kilifi

n=252

Mombasa

n=212

Approached

From HIV Clinics

Approached

**Community controls**

Kilifi

n=282

Mombasa

n=220

KHDSS, n= 145

Posters & Flyers, n= 137

Posters & Flyers

n=220

Sample analyzed

n=406

Missing outcome data**^‡^**, n=1

Missing outcome data**^‡^**, n=1

Declined to take HIV test^#^, n=3

Reactive after HIV test, n=2

**Exclusion from analysis**

Recruited sample

**n= 407**

Recruited sample

KHDSS=78 | Poster & Flyer=334

**n= 412**

Declined, n=28

unavailable, work/school

n=25

Pregnant, n=4

Relocated, n=3

Declined, n=6

Unavailable, n=65

Pregnant, n=5

Declined, n=4

Unavailable, n=6

Pregnant, n=1

**Notes**. YLWH – Young People Living With HIV/AIDS. KHDSS - Kilifi Health and Demographic Surveillance System. **^‡^** Due to a technical error on the windows tablet data capture platform, and participants could not be reached on contact details provided for reassessment. **^#^** HIV testing was the last step of community control assessments, and these participants declined to be tested even though they initially consented

This flowchart is officially published in BMC Psychiatry: Nyongesa, M.K., Mwangi, P., Kinuthia, M. et al. Prevalence, risk and protective indicators of common mental disorders among young people living with HIV compared to their uninfected peers from the Kenyan coast: a cross-sectional study. BMC Psychiatry 21, 90 (2021). https://doi.org/10.1186/s12888-021-03079-4
