# Supplementary material for: Alcohol and illicit drug use among young people living with HIV compared to their uninfected peers from the Kenyan coast: prevalence and risk indicators
Source: Subst Abuse Treat Prev Policy. 2021 Nov 24;16:86. doi: 10.1186/s13011-021-00422-6 (PMC8613997; doi:10.1186/s13011-021-00422-6)
Supplement: Supplementary file 2 — Additional file 2. HIV-related characteristics of YLWH. This additional file summarizes, in a table, the HIV-related characteristics of 406 young people living with HIV aged 18–24 years from the Kenyan coast. [file 13011_2021_422_MOESM2_ESM.docx]

**Additional File 2: HIV-related characteristics of YLWH from the Kenyan coast, n=406**

| **Characteristic** | | **Mean/Frequency** | **SD/ percent** |
| --- | --- | --- | --- |
| **Recent Body Mass Index** – kg/m^2^, mean, SD | | 20.6 | 3.6 |
| **Any current chronic illness** | |  |  |
| *No* | 399 | 98.3 |  |
| *Yes ^†^* | 7 | 1.7 |  |
| **ART regimen** | |  |  |
| *First line* | 330 | 81.3 |  |
| *Second line ^1^* | 76 | 18.7 |  |
| **Viral load** | |  |  |
| *≤1000 copies/mL* | 280 | 69.0 |  |
| *>1000 copies/mL* | 126 | 31.0 |  |
| **WHO clinical stage**, OM=1 | |  |  |
| *Stage 1* | 249 | 61.5 |  |
| *Stage 2* | 102 | 25.2 |  |
| *Stage 3* | 52 | 12.8 |  |
| *Stage 4* | 2 | 0.5 |  |
| **Duration on ART** | |  |  |
| *>5 years* | 235 | 57.9 |  |
| *1-5 years* | 124 | 30.5 |  |
| *6-11months* | 25 | 6.2 |  |
| *<6months* | 22 | 5.4 |  |
| **Any current opportunistic infection** | |  |  |
| *No* | 381 | 93.8 |  |
| *Yes* | 25 | 6.2 |  |
| **Any ART side effects**, OM=1 | |  |  |
| *No* | 296 | 73.1 |  |
| *Yes* | 109 | 26.9 |  |
| **HIV status disclosure** | |  |  |
| *Yes* | 381 | 93.8 |  |
| *No* | 25 | 6.2 |  |
| **Clinic accessibility** | |  |  |
| *Easily accessible (Less than 30 min)* | 129 | 31.8 |  |
| *Somehow accessible (30 min to 1 hour)* | 175 | 43.1 |  |
| *Not accessible (>1 hour)* | 102 | 25.1 |  |
| **Satisfaction with current care** | |  |  |
| *Satisfied* | 383 | 94.3 |  |
| *Neutral* | 16 | 3.9 |  |
| *Not satisfied* | 7 | 1.7 |  |
| **Notes.** ^†^ one or more chronic illness in addition to HIV, **OM** – observation with missing value, **WHO** – world health organization, **ART** – antiretroviral therapy, **1** - all participants were initially started on 1^st^ line ART but had changed to 2^nd^ line ART at the time of data collection | | | |
