# Supplementary material for: Alcohol and illicit drug use among young people living with HIV compared to their uninfected peers from the Kenyan coast: prevalence and risk indicators
Source: Subst Abuse Treat Prev Policy. 2021 Nov 24;16:86. doi: 10.1186/s13011-021-00422-6 (PMC8613997; doi:10.1186/s13011-021-00422-6)
Supplement: Supplementary file 3 — Additional file 3. Association between HIV-infection status and current substance use. This additional file summarizes, in a table, the association between HIV infection status and substance use (alcohol, illicit drugs, or both) among young people from the Kenyan coast. [file 13011_2021_422_MOESM3_ESM.docx]

**Additional File 3: A logistic regression analysis of the association between HIV infection status and substance use among young people from the Kenyan coast, n=812**

|  | | **Univariable analysis OR (95% CI)** | **Multivariable analysis**  **aOR (95% CI)** |  | **Univariable analysis OR (95% CI)** | **Multivariable analysis**  **aOR (95% CI)** |  | **Univariable analysis OR (95% CI)** | **Multivariable analysis**  **aOR (95% CI)** |
| --- | --- | --- | --- | --- | --- | --- | --- | --- | --- |
| **Current alcohol use** | |  |  | **Current illicit drug use** |  |  | **alcohol & illicit drug use** |  |  |
| No | Ref | Ref |  | Ref | Ref |  | Ref | Ref |  |
| Yes | 0.48*** (0.33, 0.69) | 0.47*** (0.32, 0.70) |  | 0.46** (0.29, 0.73) | 0.50** (0.31, 0.83) |  | 0.29*** (0.16, 0.55) | 0.30*** (0.16, 0.58) |  |
| **Variables adjusted for:** | |  |  |  |  |  |  |  |  |
| **Sex** | |  |  |  |  |  |  |  |  |
| Female |  | Ref |  |  | Ref |  |  | Ref |  |
| Male |  | 2.68*** (1.79, 4.01) |  |  | 7.33*** (3.87, 13.88) |  |  | 7.00*** (3.19, 15.35) |  |
| **Area of residence** | |  |  |  |  |  |  |  |  |
| Rural (Kilifi) |  | Ref |  |  | Ref |  |  | Ref |  |
| Urban (Mombasa) |  | 1.21 (0.80, 1.81) |  |  | 1.72* (1.01, 2.92) |  |  | 0.93 (0.50, 1.74) |  |
| **Asset index** | |  | 1.28*** (1.13, 1.44) |  |  | 1.19* (1.02, 1.39) |  |  | 1.37** (1.14, 1.65) |
| **Religion** | |  |  |  |  |  |  |  |  |
| *Muslim* |  | Ref |  |  | 1.34 (0.78, 2.29) |  |  | 0.97 (0.48, 1.94) |  |
| *Christian* |  | 2.73*** (1.58, 4.71) |  |  | Ref |  |  | Ref |  |
| *No religion* |  | 2.47 (0.95, 6.39) |  |  | 2.21 (0.88, 5.53) |  |  | 1.90 (0.67, 5.34) |  |
| **Negative life events** | |  |  |  |  |  |  |  |  |
| None |  | Ref |  |  | Ref |  |  | Ref |  |
| 1-5 events |  | 1.56 (0.74, 3.28) |  |  | 3.64* (1.07, 12.41) |  |  | 7.15 (0.93, 55.23) |  |
| 6+ events |  | 4.32*** (2.00, 9.37) |  |  | 6.52** (1.85, 22.98) |  |  | 22.18** (2.81, 175.35) |  |
| **n** | |  | 812 |  |  | 812 |  |  | 812 |
| **R^2^** | |  | 12.1% |  |  | 17.0% |  |  | 19.7% |
| **Notes**. ***** p value<0.05, ****** p value <0.01, *** p value <0.001  **OR**- unadjusted odds ratio, **aOR**- adjusted odds ratio, **Ref-** reference group **R^2^** – variance explained by the model, as a percentage | | | | | | | | | |

**Association between HIV infection status and current substance use**

|  | | **Univariable analysis OR (95% CI)** | **Multivariable analysis**  **aOR (95% CI)** |  | **Univariable analysis OR (95% CI)** | **Multivariable analysis**  **aOR (95% CI)** |  | **Univariable analysis OR (95% CI)** | **Multivariable analysis**  **aOR (95% CI)** |
| --- | --- | --- | --- | --- | --- | --- | --- | --- | --- |
| ***Hazardous* alcohol use** | |  |  | ***Hazardous* illicit drug use** |  |  | **alcohol & illicit drug use** |  |  |
| No | Ref | Ref |  | Ref | Ref |  | Ref | Ref |  |
| Yes | 0.74 (0.42, 1.32) | 0.59 (0.31, 1.13) |  | 0.69 (0.40, 1.18) | 0.59 (0.32, 1.10) |  | 0.76 (0.33, 1.76) | 0.78 (0.29, 2.06) |  |
| **Variables adjusted for:** | |  |  |  |  |  |  |  |  |
| **Age** | |  | 1.10 (0.93, 1.29) |  |  | 0.92 (0.78, 1.08) |  |  | 1.10 (0.86, 1.41) |
| **Sex** | |  |  |  |  |  |  |  |  |
| Female |  | Ref |  |  | Ref |  |  | Ref |  |
| Male |  | 2.26* (1.17, 4.38) |  |  | 5.51*** (2.69, 11.28) |  |  | 8.15*** (2.50, 26.55) |  |
| **Area of residence** | |  |  |  |  |  |  |  |  |
| Rural (Kilifi) |  | Ref |  |  | Ref |  |  | Ref |  |
| Urban (Mombasa) |  | 0.72 (0.36, 1.43) |  |  | 2.26* (1.12, 4.55) |  |  | 0.64 (0.23, 1.78) |  |
| **Asset index** | |  | 1.34** (1.09, 1.64) |  |  | 1.18 (0.97, 1.43) |  |  | 1.60** (1.17, 2.19) |
| **Education** | |  |  |  |  |  |  |  |  |
| Secondary |  | Ref |  |  | Ref |  |  | Ref |  |
| Tertiary |  | 0.87 (0.33, 2.26) |  |  | 1.31 (0.58, 2.92) |  |  | 0.74 (0.17, 3.33) |  |
| Primary |  | 2.29* (1.07, 4.89) |  |  | 1.49 (0.68, 3.24) |  |  | 1.94 (0.64, 5.89) |  |
| None**†** |  | 2.08 (0.22, 19.59) |  |  | 1.00 |  |  | 1.00 |  |
| **Employment** | |  |  |  |  |  |  |  |  |
| Student |  | Ref |  |  | Ref |  |  | Ref |  |
| Self-employed |  | 2.77 (0.94, 8.19) |  |  | 1.36 (0.43, 4.29) |  |  | 6.30* (1.15, 34.36) |  |
| Formally employed**†** |  | 2.41 (0.53, 10.87) |  |  | 0.98 (0.11, 8.38) |  |  | 1.00 |  |
| Unemployed |  | 1.72 (0.69, 4.31) |  |  | 2.20* (1.05, 4.60) |  |  | 3.21 (0.72, 14.35) |  |
| **Living arrangement** | |  |  |  |  |  |  |  |  |
| Family/Relative |  | Ref |  |  | Ref |  |  | Ref |  |
| Friend/non-relative |  | 4.07 (0.98, 16.99) |  |  | 1.45 (0.28, 7.49) |  |  | 7.19* (1.11,46.43) |  |
| Alone |  | 2.64* (1.05, 6.60) |  |  | 0.57 (0.18, 1.80) |  |  | 1.28 (0.24, 6.75) |  |
| **Negative life events** | |  |  |  |  |  |  |  |  |
| None**†** |  | Ref |  |  | Ref |  |  | 1.00 |  |
| 1-5 events |  | 3.16 (0.40, 24.75) |  |  | 4.56 (0.59, 34.97) |  |  | Ref |  |
| 6+ events |  | 9.96* (1.27, 77.82) |  |  | 9.84* (1.26, 77.03) |  |  | 5.10** (1.85, 14.08) |  |
| ***Emotional problems*** | |  |  |  |  |  |  |  |  |
| *Not present* |  | Ref |  |  | Ref |  |  | Ref |  |
| *Present* |  | 2.22 (0.99, 5.00) |  |  | 3.12** (1.39, 7.02) |  |  | 4.77** (1.57, 14.45) |  |
| **n** | |  | 812 |  |  | 800 |  |  | 698 |
| **R^2^** | |  | 14.9% |  |  | 16.2% |  |  | 25.0% |
| **Notes**. ***** p value<0.05, ****** p value <0.01, *** p value <0.001  **†** - for these variable categories no participant currently used any illicit drug or together with alcohol, hence there was perfect prediction of failure in logistic regression analyses denoted by the null value of 1.00  **OR**- unadjusted odds ratio, **aOR**- adjusted odds ratio, **Ref-** reference group **R^2^** – variance explained by the model, as a percentage | | | | | | | | | |

**Association between HIV infection status and *hazardous* substance use**
