# Supplementary material for: Alcohol and illicit drug use among young people living with HIV compared to their uninfected peers from the Kenyan coast: prevalence and risk indicators
Source: Subst Abuse Treat Prev Policy. 2021 Nov 24;16:86. doi: 10.1186/s13011-021-00422-6 (PMC8613997; doi:10.1186/s13011-021-00422-6)
Supplement: Supplementary file 4 — Additional file 4. Univariate analysis of risk indicators for current substance use among young people. This additional file summarizes, in a table, results from univariable logistic regression analyses of the risk indicators for current alcohol use, illicit drug use, or both, among young people from the Kenyan coast. [file 13011_2021_422_MOESM4_ESM.docx]

**Additional File 4: Univariate logistic regression analysis of risk indicators for any current substance use among young people from the Kenyan Coast**

|  | | **Current use of alcohol** | | | **Current use of illicit drugs** | | | **Current alcohol and illicit drug use comorbidity** | | |
| --- | --- | --- | --- | --- | --- | --- | --- | --- | --- | --- |
|  | | **Whole sample, N=812** | **YLWH, n=406** | **HIV uninfected, n=406** | **Whole sample, N=812** | **YLWH, n=406** | **HIV uninfected, n=406** | **Whole sample, N=812** | **YLWH, n=406** | **HIV uninfected, n=406** |
| **Covariate** | | **OR (95% CI)** | **OR (95% CI)** | **OR (95% CI)** | **OR (95% CI)** | **OR (95% CI)** | **OR (95% CI)** | **OR (95% CI)** | **OR (95% CI)** | **OR (95% CI)** |
| **Age** | | 1.11** (1.01, 1.21) | 1.06 (0.93, 1.21) | 1.13** (1.01, 1.28) | 1.00 (0.90, 1.11) | 0.96 (0.81, 1.14) | 1.01 (0.87, 1.16) | 1.08 (0.94, 1.23) | 1.16 (0.90, 1.48) | 1.03 (0.87, 1.21) |
| **Sex** | |  |  |  |  |  |  |  |  |  |
| Female | Ref | Ref | Ref | Ref | Ref | Ref | Ref | Ref | Ref |  |
| Male | 2.60*** (1.78, 3.78) | 2.02** (1.12, 3.63) | 2.78*** (1.69, 4.60) | 8.07*** (4.32, 15.09) | 5.89*** (2.35, 14.76) | 9.32*** (3.91, 22.23) | 7.21*** (3.37, 15.43) | 3.40** (1.05, 11.04) | 9.67*** (3.39, 27.60) |  |
| **Area of residence** | |  |  |  |  |  |  |  |  |  |
| Rural (Kilifi) | Ref | Ref | Ref | Ref | Ref | Ref | Ref | Ref | Ref |  |
| Urban (Mombasa) | 1.46** (1.02, 2.09) | 1.24 (0.70, 2.22) | 1.62** (1.02, 2.57) | 2.14*** (1.35, 3.39) | 3.58*** (1.50, 8.54) | 1.68* (0.96, 2.95) | 1.33 (0.77, 2.27) | 1.84 (0.60, 5.58) | 1.19 (0.63, 2.22) |  |
| **Religion** | | Overall p=0.03 | Overall p=0.06 | Overall p=0.12 | Overall p=0.02 | Overall p=0.12 | Overall p=0.14 | Overall p=0.15 | Overall p=0.54 | Overall p=0.46 |
| Muslim | Ref | Ref | Ref | 1.77** (1.07, 2.92) | 2.32** (1.05, 5.11) | 1.52 (0.79, 2.93) | 1.20 (0.63, 2.31) | 1.59 (0.48, 5.29) | 1.09 (0.50, 2.41) |  |
| Christian | 2.01*** (1.20, 3.36) | 2.83** (1.09, 7.36) | 1.71* (0.91, 3.21) | Ref | Ref | Ref | Ref | Ref | Ref |  |
| No religion | 2.22* (0.92, 5.35) | 4.92* (1.02, 23.76) | 1.32 (0.45, 3.86) | 2.36** (1.04, 5.37) | 1.33 (0.16, 10.83) | 2.26* (0.90, 5.68) | 2.47* (0.98, 6.24) | 2.75 (0.32, 23.49) | 1.93 (0.68, 5.44) |  |
| **Education** | | Overall p<0.01 | Overall p=0.03 | Overall p=0.02 | Overall p=0.53 | Overall p=0.75 | Overall p=0.74 | Overall p=0.14 | Overall p=0.40 | Overall p=0.34 |
| *Secondary* | Ref | Ref | Ref | Ref | Ref | Ref | Ref | Ref | Ref |  |
| *Tertiary* | 2.38*** (1.56, 3.64) | 2.29** (1.10, 4.79) | 2.19*** (1.29, 3.73) | 1.33 (0.78, 2.28) | 0.92 (0.29, 2.97) | 1.25 (0.67, 2.34) | 1.72* (0.89, 3.33) | 0.55 (0.06, 4.79) | 1.64 (0.79, 3.43) |  |
| *Primary* | 1.00 (0.64, 1.58) | 0.87 (0.44, 1.72) | 1.34 (0.72, 2.49) | 1.02 (0.61, 1.73) | 1.30 (0.58, 2.91) | 1.01 (0.49, 2.06) | 1.43 (0.75, 2.72) | 1.79 (0.57, 5.59) | 1.62 (0.72, 3.62) |  |
| *None***†** | 1.14 (0.24, 5.33) | 1.00 | 4.59* (0.62, 33.84) | 1.00 | 1.00 | 1.00 | 1.00 | 1.00 | 1.00 |  |
| **Employment** | | Overall p=0.57 | Overall p=0.92 | Overall p=0.42 | Overall p=0.77 | Overall p=0.77 | Overall p=0.71 | Overall p=0.61 | Overall p=0.13 | Overall p=0.84 |
| Student | Ref | Ref | Ref | Ref | Ref | Ref | Ref | Ref | Ref |  |
| Self-employed | 1.33 (0.75, 2.38) | 0.92 (0.32, 2.66) | 1.58 (0.78, 3.23) | 0.79 (0.35, 1.77) | 1.28 (0.32, 5.05) | 0.61 (0.22, 1.69) | 1.27 (0.52, 33.15) | 7.00* (0.62, 79.20) | 0.91 (0.32, 2.59) |  |
| Formally employed | 1.73 (0.69, 4.30) | 1.58 (0.41, 6.08) | 2.23 (0.62, 8.09) | 1.06 (0.30, 3.71) | 1.11 (0.13, 9.48) | 1.28 (0.26, 6.28) | 0.61 (0.08, 4.75) | 9.33* (0.55, 156.97) | 1.00 |  |
| Unemployed | 1.13 (0.76, 1.67) | 1.03 (0.54, 1.94) | 1.29 (0.78, 2.15) | 1.17 (0.72, 1.88) | 1.58 (0.67, 3.75) | 1.10 (0.61, 1.97) | 1.42 (0.78, 2.59) | 7.11* (0.90, 56.15) | 1.18 (0.60, 2.31) |  |
| **Relationship status** | | Overall p=0.34 | Overall p=0.45 | Overall p=1.00 | Overall p=0.08 | Overall p=0.10 | Overall p=0.72 | Overall p=0.68 | Overall p=0.69 | Overall p=0.67 |
| Never married | 1.19 (0.69, 2.05) | 1.08, 0.50, 2.34 | 1.02 (0.46, 2.24) | 2.04* (0.92, 4.54) | 3.35* (0.78, 14.42) | 1.20 (0.45, 3.20) | 1.19 (0.52, 2.69) | 1.36 (0.30, 6.22) | 0.81 (0.30, 2.19) |  |
| *Separated***†** | 0.52 (0.14, 1.88) | 0.29 (0.04, 2.46) | 1.07 () 0.18, 6.28 | 1.00 | 1.00 | 1.00 | 1.00 | 1.00 | 1.00 |  |
| *Married/cohabiting* | Ref | Ref | Ref | Ref | Ref | Ref | Ref | Ref | Ref |  |
| **Living arrangement** | | Overall p<0.01 | Overall p=0.06 | Overall p=0.04 | Overall p=0.58 | Overall p=0.14 | Overall p=0.71 | Overall p=0.34 | Overall p=0.02 | Overall p=0.67 |
| Family/Relative | Ref | Ref | Ref | Ref | Ref | Ref | Ref | Ref | Ref |  |
| Friend/non-relative**†** | 1.52 (0.49, 4.75) | 2.17 (0.44, 10.79) | 1.18 (0.23, 5.97) | 1.11 (0.25, 4.96) | 1.00 | 1.97 (0.39, 10.01) | 1.87 (0.41, 8.40) | 1.00 | 2.82 (0.55, 14.45) |  |
| Alone | 2.48*** (1.47, 4.16) | 2.53** (1.11, 5.75) | 2.47** (1.24, 4.89) | 1.44 (0.73, 2.84) | 2.17* (0.78, 6.06) | 1.07 (0.43, 2.69) | 1.67 (0.76, 3.68) | 4.39** (1.30, 14.78) | 0.97 (0.33, 2.86) |  |
| **Asset index** | | 1.27*** (1.14, 1.42) | 1.16* (0.97, 1.38) | 1.32*** (1.14, 1.53) | 1.25*** (1.09, 1.43) | 0.94 (0.74, 1.19) | 1.44*** (1.20, 1.72) | 1.33*** (1.13, 1.57) | 0.95 (0.68, 1.34) | 1.47*** (1.19, 1.80) |
| **Negative life events** | | Overall p<0.01 | Overall p=0.25 | Overall p<0.01 | Overall p=0.06 | Overall p=0.32 | Overall p=0.08 | Overall p=0.01 | Overall p=0.01 | Overall p=0.03 |
| None**†** | Ref | Ref | Ref | Ref | 1.00 | Ref | Ref | 1.00 | Ref |  |
| 1-5 events | 1.27 (0.62, 2.57) | 0.91 (0.25, 3.26) | 1.61 (0.69, 3.78) | 3.15* (0.96, 10.33) | Ref | 2.72* (0.80, 9.17) | 5.13* (0.69, 38.17) | Ref | 5.68* (0.75, 42.82) |  |
| 6+ events | 2.48** (1.21, 5.09) | 1.98 (0.56, 6.99) | 3.85*** (1.58, 9.42) | 4.06** (1.22, 13.59) | 1.46 (0.69, 3.08) | 4.00** (1.13, 14.17) | 9.70** (1.30, 72.48) | 5.51** (1.51, 20.10) | 10.24** (1.32, 79.32) |  |
| **Parent living status** | | Overall p=0.98 | Overall p=0.47 | Overall p=0.06 | Overall p=0.05 | Overall p=0.65 | Overall p=0.01 | Overall p=0.10 | Overall p=0.41 | Overall p=0.01 |
| Both alive | Ref | Ref | Ref | 2.26** (1.04, 4.89) | 1.55 (0.60, 3.99) | Ref | 2.41* (0.92, 6.28) | 1.33 (0.39, 4.46) | Ref |  |
| One alive | 0.99 (0.66, 1.49) | 0.88 (0.42, 1.85) | 1.74** (1.02, 2.96) | 2.66** (1.19, 5.95) | 1.20 (0.47, 3.08) | 2.26*** (1.25, 4.11) | 2.43* (0.89, 6.65) | 0.51 (0.12, 2.17) | 2.32** (1.19, 4.52) |  |
| Both dead**†** | 0.95 (0.58, 1.55) | 1.34 (0.65, 2.73) | 2.34* (0.74, 7.38) | Ref | Ref | 1.00 | Ref | Ref | 1.00 |  |
| **Currently smoking** | |  |  |  |  |  |  |  |  |  |
| No | Ref | Ref | Ref | – | – | – | – | – | – |  |
| Yes | 10.68*** (5.76, 19.81) | 5.53*** (2.11, 14.47) | 17.48*** (6.91, 44.18) | – | – | – | – | – | – |  |
| **Current khat use** | |  |  |  |  |  |  |  |  |  |
| No | Ref | Ref | Ref | – | – | – | – | – | – |  |
| Yes | 10.81*** (6.40, 18.24) | 11.43*** (5.21, 25.10) | 10.52*** (5.11, 21.67) | – | – | – | – | – | – |  |
| **Current alcohol use** | |  |  |  |  |  |  |  |  |  |
| No | – | – | – | Ref | Ref | Ref | – | – | – |  |
| Yes | – | – | – | 12.41*** (7.65, 20.14) | 7.56*** (3.43, 16.66) | 15.20*** (8.00, 28.91) | – | – | – |  |
| **Emotional problems ^#^** | |  |  |  |  |  |  |  |  |  |
| Not present | | Ref | Ref | Ref | Ref | Ref | Ref | Ref | Ref | Ref |
| Present | | 1.33 (0.77, 2.30) | 1.88* (0.94, 3.76) | 1.50 (0.55, 4.06) | 1.39 (0.72, 2.67) | 1.34 (0.53, 3.43) | 2.85** (1.04, 7.81) | 1.43 (0.65, 3.12) | 2.17 (0.66, 7.14) | 2.31 (0.73, 7.31) |
| **Opportunistic infection** | |  |  |  |  |  |  |  |  |  |
| No | NA | Ref | NA | NA | Ref | NA | NA | Ref | NA |  |
| Yes | NA | 2.24* (0.85, 5.90) | NA | NA | 1.79 (0.50, 6.35) | NA | NA | 4.59** (1.19, 17.64) | NA |  |
| **ART duration** | |  | Overall p=0.76 |  |  | Overall p=0.90 |  |  | Overall p=0.XX |  |
| >5 years | NA | Ref | NA | NA | Ref | NA | NA | Ref | NA |  |
| 1-5 years | NA | 0.69 (0.35, 1.37) | NA | NA | 0.77 (0.31, 1.90) | NA | NA | 0.62 (0.17, 2.34) | NA |  |
| 6-11 months | NA | 0.81 (0.23, 2.84) | NA | NA | 1.75 (0.47, 6.44) | NA | NA | 1.05 (0.13, 8.62) | NA |  |
| <6 months | NA | 0.93 (0.26, 3.33) | NA | NA | 2.02 (0.54, 7.53) | NA | NA | 1.20 (0.14, 9.90) | NA |  |
| **Clinic accessibility** | |  | Overall p=0.29 |  |  | Overall p=0.55 |  |  | Overall p=0.XX |  |
| Inaccessible | NA | 0.83 (0.35, 1.93) | NA | NA | 0.83 (0.33, 2.11) | NA | NA | 0.41 (0.08, 2.08) | NA |  |
| Somehow accessible | NA | 1.45 (0.74, 2.84) | NA | NA | 0.59 (0.25, 1.41) | NA | NA | 0.73 (0.23, 2.31) | NA |  |
| Easily accessible | NA | Ref | NA | NA | Ref | NA | NA | Ref | NA |  |
| **Satisfaction with care** | |  | Overall p=0.39 |  |  | Overall p=0.78 |  |  | Overall p=0.12 |  |
| Satisfied | NA | Ref | NA | NA | Ref | NA | NA | Ref | NA |  |
| Neutral | NA | 1.61 (0.44, 5.86) | NA | NA | 0.85 (0.11, 6.63) | NA | NA | 2.06 (0.25, 16.90) | NA |  |
| Not satisfied | NA | 2.79 (0.53, 14.79) | NA | NA | 2.11 (0.25, 18.17) | NA | NA | 5.15* (0.57, 46.21) | NA |  |
| **Viral load** | |  |  |  |  |  |  |  |  |  |
| ≤1000 copies/mL | NA | Ref | NA | NA | Ref | NA | NA | Ref | NA |  |
| >1000 copies/mL | NA | 1.55* (0.85, 2.81) | NA | NA | 1.12 (0.51, 2.47) | NA | NA | 1.24 (0.41, 3.79) | NA |  |
| **Current chronic illness** | |  |  |  |  |  |  |  |  |  |
| *No* | NA | Ref | NA | NA | Ref | NA | NA | Ref | NA |  |
| *Yes* | NA | 1.11 (0.13, 9.42) | NA | NA | 2.13 (0.25, 18.26) | NA | NA | 1.00 | NA |  |
| **ART regimen** | |  |  |  |  |  |  |  |  |  |
| *First line* | NA | Ref | NA | NA | Ref | NA | NA | Ref | NA |  |
| *Second line* | NA | 1.32 (0.66, 2.66) | NA | NA | 1.65 (0.70, 3.86) | NA | NA | 2.51* (0.82, 7.72) | NA |  |
| **WHO clinical stage** | |  |  |  |  |  |  |  |  |  |
| *Stage 1 & 2* | NA | Ref | NA | NA | Ref | NA | NA | Ref | NA |  |
| *Stage 3 & 4* | NA | 0.81 (0.33, 1.99) | NA | NA | 1.70 (0.66, 4.38) | NA | NA | 1.09 (0.24, 4.99) | NA |  |
| **ART side effects** | |  |  |  |  |  |  |  |  |  |
| *No* | NA | Ref | NA | NA | Ref | NA | NA | Ref | NA |  |
| *Yes* | NA | 1.33 (0.72, 2.49) | NA | NA | 0.81 (0.34, 1.96) | NA | NA | 0.73 (0.20, 2.68) | NA |  |
| **HIV status disclosure** | |  |  |  |  |  |  |  |  |  |
| *Yes* | NA | Ref | NA | NA | Ref | NA | NA | Ref | NA |  |
| *No* | NA | 0.26 (0.03, 1.99) | NA | NA | 1.00 | NA | NA | 1.00 | NA |  |
| **Notes.** * p value<0.15, ** p value <0.05, *** p value <0.01  Only variables with p-value <0.15 in the univariate or p< 0.05 in the multivariable analysis are presented here  # co-occurrence of both depressive and anxiety symptoms  † - for these variable categories, no participant currently used alcohol, illicit drugs or both, hence there was perfect prediction of failure in the regression analyses denoted by null value of 1.00  – variable not included in analysis  **OR**- odds ratio, **aOR**- adjusted odds ratio, **Ref**- reference group, **ART**- antiretroviral therapy **NA**- not applicable | | | | | | | | | | |
